# Fundamento y generalidades de la construcción de modelos estadísticos multinivel en el ámbito de la investigación en salud

**DOI:** 10.7705/biomedica.6946

**Published:** 2023-12-01

**Authors:** Andry Yasmid Mera-Mamián, José Moreno-Montoya, Laura Andrea Rodriguez-Villamizar, Diana Isabel Muñoz, Ángela María Segura, Héctor Iván García

**Affiliations:** 1 Escuela de Graduados, Universidad CES, Medellín, Colombia Universidad CES Universidad CES Medellín Colombia; 2 División de Estudios Clínicos y Epidemiología Clínica, Hospital Universitario de la Fundación Santa Fe de Bogotá, Bogotá, D.C., Colombia Hospital Universitario de la Fundación Santa Fe de Bogotá Bogotá D.C Colombia; 3 Departamento de Salud Pública, Escuela de Medicina, Universidad Industrial de Santander, Bucaramanga, Colombia Universidad Industrial de Santander Universidad Industrial de Santander Bucaramanga Colombia; 4 Facultad de Fisioterapia, Universidad CES, Medellín, Colombia Universidad CES Universidad CES Medellín Colombia; 5 Instituto de Cancerología Las Américas, Medellín, Colombia Instituto de Cancerología Las Américas Medellín Colombia

**Keywords:** análisis multinivel, investigación sobre servicios de salud, bioestadística, sesgo, multilevel analysis, health services research, biostatistics, bias

## Abstract

Este trabajo tiene como objetivo presentar una mirada global de la aplicabilidad de los modelos de análisis multinivel en el ámbito de la investigación sanitaria. Ofrece información sobre los fundamentos teóricos, metodológicos y estadísticos y, además, menciona los pasos básicos para la construcción de estos modelos, y da ejemplos de su uso, según la estructura jerárquica de los datos.

Cabe resaltar que, antes de utilizar estos modelos, se requiere contar con un soporte teórico sobre la necesidad de uso y una valoración estadística que dé cuenta del porcentaje de varianza explicada por el efecto de agrupación de las observaciones. Los requisitos para llevar a cabo este tipo de análisis dependen de condiciones especiales como el tipo de variables, la cantidad de unidades por nivel o el tipo de estructura jerárquica. Se concluye que los modelos de análisis multinivel son una herramienta útil para lograr la integración de información, dadas la complejidad de las relaciones y las interacciones que determinan la mayoría de las condiciones de salud, incluida la pérdida de independencia entre las unidades de observación.

## Abstract (en)

This topic review aims to present a global vision of multilevel analysis models' applicability to health research, explaining its theoretical, methodological, and statistical foundations. We describe the basic steps to build these models and examples of their application according to the data hierarchical structure.

It ir worth noticing that before using these models, researchers must have a rationale for needing them, and a statistical evaluation accounting for the variance percentage explained by the observations grouping effect.

The requirements to conduct this type of analysis depends on special conditions such as the type of variables, the number of units per level, or the type of hierarchical structure. We conclude that multilevel analysis models are a useful tool to integrate information, considering the complexity of the relationships and interactions involved in most health conditions, including the loss of independence between observation units.

En investigación sanitaria, las estructuras jerárquicas o anidadas aparecen cuando las unidades de observación en las que se mide el resultado de interés, se asocian naturalmente dentro de otras que las agrupan. Esto sucede, por ejemplo, con individuos humanos que se agrupan en unidades superiores, como familias, barrios o escuelas ^1^. La misma agrupación puede encontrarse al considerar como unidades de observación el conjunto de mediciones repetidas en el tiempo para un mismo sujeto ^2^, o cuando las observaciones se recopilan con metodologías cuya selección se basa en el emparejamiento ^3^.

Dada la frecuencia con la que el fenómeno ocurre en el ámbito de la salud, aprovechar la estructura jerarquizada puede resultar útil para ampliar el alcance de la investigación científica, a la vez que mejora la comprensión del efecto que exposiciones a diferentes niveles pueden provocar en los individuos. No obstante, el uso de modelos para datos jerarquizados debe obedecer a una necesidad analítica prestablecida desde la formulación de la pregunta y la hipótesis de trabajo. En ese sentido, es necesario que el supuesto de una asociación de interés entre una o varias exposiciones y un resultado específico (dada la agrupación de las unidades de análisis), se encuentre biológica, social o clínicamente soportado.

Una forma de evaluar estadísticamente si existe un efecto de agrupación entre las unidades de análisis, es el coeficiente de correlación intraclase *(intraclass correlation coefficient,* ICC), una medida que hace referencia a la fuerza de la asociación lineal existente entre las mediciones del resultado de interés de los sujetos individuales, una vez comparada con la que se deriva de la comparación entre grupos ^4^.

Los análisis multinivel surgen en el área de la psicometría y la educación como una alternativa para explorar el efecto que tienen las estructuras de relaciones jerárquicas o de datos anidados en la valoración de las exposiciones y los resultados de interés en el ámbito epidemiológico, y consideran la pérdida de independencia de los sujetos de observación debido a su cercanía en cada nicho agrupador. Estos, al igual que los modelos de regresión de un único nivel, dependen de la escala de la variable respuesta y del tipo de función de enlace usado en su construcción para su especificación ^1^.

Este tipo de análisis aporta en el control de la denominada "falacia ecológica" o sesgo de agregación, que se produce cuando se infiere inadecuadamente la variabilidad interindividual a partir de información captada a nivel grupal, cuando las variables individuales y grupales tienen efectos independientes o miden conceptos teóricos diferentes ^5^; esto puede generar que se asuma erróneamente que la correlación estadística entre dos variables a nivel agregado es igual a la correlación entre las correspondientes variables a nivel individual ^6^.

Por lo tanto, considerando su aporte en el análisis de datos anidados en diferentes escenarios, el presente trabajo tiene como propósito ofrecer una mirada global de la construcción, el uso y las particularidades metodológicas de este tipo de modelos en el ámbito de la investigación sanitaria.

## Fundamento del uso de análisis multinivel

La falta de independencia entre las unidades de observación respecto a la oportunidad de considerarlas expuestas o en riesgo, constituye un desafío común en la investigación en salud y su omisión puede implicar la sobrestimación de la significación estadística ^7^^,^^8^ y de los tamaños de efecto ^9^, lo que lleva a la obtención de estimaciones con intervalos de confianza inapropiadamente estrechos y al aumento del riesgo de error de tipo I ^10^. Para enfrentar el problema, los modelos multinivel toman en cuenta el nivel de agregación de las unidades de observación ^11^^,^^12^.

El análisis multinivel puede adaptarse a una estructura jerárquica clásica y a estructuras con jerarquías y relaciones complejas, como la clasificación cruzada o la membresía múltiple. La estructura jerárquica clásica se presenta cuando unidades de nivel 1 están anidadas en grupos que, a su vez, pueden pertenecer a otros de nivel superior, como individuos anidados en escuelas que, a su vez, se anidan en barrios, o el caso de los metaanálisis, en los que el resultado se mide de forma individual o en grupo mediante la medida de resumen obtenida para cada estudio ^2^. También, ocurre con las medidas repetidas, ya que diferentes mediciones a lo largo del tiempo pueden provenir del mismo individuo, quien representa un nivel superior ^13^^,^^14^.

Por su parte, el análisis de clasificación cruzada se presenta cuando unidades de nivel inferior corresponden a más de un nivel superior a la vez, sin que estos dos niveles superiores estén anidados entre sí ^15^. En el caso de la estructura no jerárquica de membresía múltiple, las unidades del primer nivel pueden pertenecer a varias unidades del nivel superior. Por ejemplo, cuando se analizan datos de individuos relacionados con su lugar de estudio en un periodo específico, en el que pudieron asistir a diferentes escuelas ^4^.

En los modelos multinivel, al igual que en los modelos convencionales de regresión (de un solo nivel), también son importantes los términos *intercepto* (valor de la variable dependiente cuando las variables explicativas valen 0) y *pendiente* (cambio en el valor de la variable dependiente por unidad de cambio en una variable independiente, una vez consideradas constantes las demás). No obstante, cuando se trata de datos agrupados, la intersección y la pendiente pueden variar en cada grupo según el nicho agrupador de las observaciones o la magnitud de una variable medida a ese nivel, lo que convierte en un tema de interés la estimación de dichos parámetros y su variación en los modelos multinivel.

De acuerdo con lo anterior, es posible construir dos tipos de modelos principales: de interceptos aleatorios (también denominados de efectos fijos) y de efectos aleatorios (también denominados de pendientes aleatorias). En el primero, el término de intersección varía aleatoriamente entre los grupos, pero los efectos (pendientes) son los mismos. En este caso, la variable dependiente se ve afectada por un componente fijo que corresponde a la media general del intercepto común en todos los conglomerados y los efectos de las variables independientes (también comunes), además del componente aleatorio: la variación entre individuos y la variación del intercepto entre los conglomerados ^16^. Este tipo de modelos resulta de particular utilidad cuando se requiere valorar en qué medida las diferencias entre individuos se deben a su pertenencia a grupos después de ajustarlos por otras variables ^17^.

**Figura 1:**
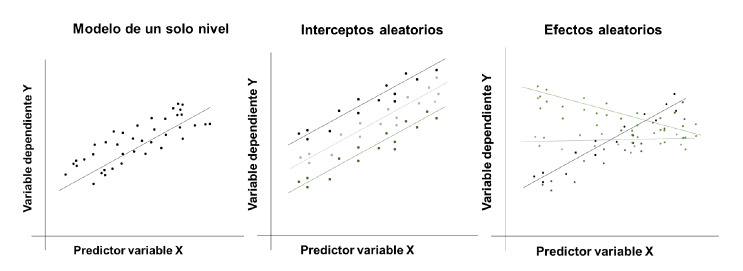
Comparación entre modelos de un solo nivel, interceptos aleatorios y efectos aleatorios

En el segundo modelo, tanto interceptos como efectos-pendientes son aleatorios ^2^, lo que permite a las unidades de observación tener sus propias trayectorias, aspecto importante cuando los grupos de observación tienen características muy heterogéneas. A diferencia del modelo de efectos fijos, es razonable considerar que el efecto a nivel de grupo varía aleatoriamente entre estos, es decir, los efectos de las variables explicativas varían entre grupos ^17^. En la figura 1, se compara un modelo de regresión de un solo nivel, uno de interceptos aleatorios y otro de efectos aleatorios.

La elección de uno u otro modelo depende de la pregunta de investigación, los objetivos del estudio, la escala de medida de la variable usada para conformar los grupos (discreta o continua) y el contexto ^1^.

Respecto al método de estimación de los coeficientes, los más utilizados para la regresión multinivel se basan en la máxima verosimilitud, en particular, el método de máxima verosimilitud restringida o residual que emplea una función de probabilidad calculada a partir de un conjunto transformado de datos cuando hay muchos parámetros no especificados o desconocidos. Esto ayuda a obtener estimadores menos sesgados de varianza y covarianza, y tiene la ventaja de que se puede usar para dar estimadores con datos no normales ^18^.

Este método permite el uso de predictores o variables independientes en todos los niveles de análisis debido a que se crean ecuaciones de predicción para cada nivel de la estructura anidada, y se presentan ventajas sobre otros modelos cuando existe una variación del parámetro de pendiente en el nivel superior. Al respecto, se ha indicado que, ante la sospecha de endogeneidad (falta de correlación entre los residuos dentro del grupo), se debería considerar el uso de modelos de un solo nivel. Cuando los tamaños de muestra de nivel superior son relativamente pequeños, la estimación bayesiana basada en el algoritmo de cadenas de Markov con integración Monte Carlo puede tener ventaja sobre el método de máxima verosimilitud restringida o residual, si las probabilidades previas se especifican correctamente ^19^.

En paralelo, los modelos multinivel, al igual que los modelos de regresión convencionales, se pueden aplicar en cualquier variable que pueda ser modelada con un modelo lineal generalizado ^14^. Tal como ocurre en los modelos de un solo nivel, cuando no es posible asumir que la distribución de errores se corresponde con una normal, se utiliza una función de enlace para transformar el predictor. Por ejemplo, si la variable dependiente es dicotómica o se utilizan datos agregados como proporciones, se sugiere utilizar modelos de desenlace binario *(logit, probit* o doble *log* complementario) y se modela una transformación no lineal de la probabilidad de estar en una u otra categoría. En caso de que la variable respuesta tenga más de dos categorías, pueden considerarse otros modelos como el *logit* multinomial y el modelo de probabilidades proporcionales *(logit* acumulativo). Si se trata de conteo, cuando el tamaño de la población es grande o el evento es raro, se prefiere aplicar el modelo Poisson. Pueden considerarse modelos análogos para situaciones en las que se requiere valorar la supervivencia o el tiempo transcurrido hasta la ocurrencia de un evento determinado ^4^.

Como ejemplo, se cita a continuación el trabajo de Webster *et al.* ^20^, en el que, mediante ecuaciones de estimación generalizadas con función de enlace *logit,* se evaluó de manera conjunta la influencia del nivel socioeconómico individual y comunitario sobre el riesgo de padecer cáncer de mama. Se construyeron tres modelos: en el primero se consideró el nivel socioeconómico individual, en el segundo, solo el nivel comunitario, y el tercero correspondió a un análisis multinivel que incluyó ambos. Los resultados indican que las mujeres que vivían en comunidades con un nivel socioeconómico más alto tenían un riesgo mayor de desarrollar cáncer de mama, independientemente de su propio nivel socioeconómico *(Odds Ratio,* OR=1,30). Concluyeron que era posible que la medida a nivel comunitario capturara un aspecto no medido del nivel socioeconómico individual.

En cuanto a la medida del efecto contextual general, que señala la proporción de la varianza individual total atribuible al nivel de grupo, en modelos de regresión lineal multinivel, se puede calcular a partir del coeficiente de partición de varianza (CPV) ^21^. Cuando los resultados son de naturaleza binaria, corresponden al tiempo trascurrido hasta el evento o son recuentos enteros que denotan el número de veces que ocurrió un evento; el efecto contextual general se puede cuantificar mediante medidas de heterogeneidad, como la mediana de la *Odds Ratio (Median Odds Ratio,* MOR), la mediana de la razón de riesgos *(Median Hazard Ratio,* MHR) o la mediana de la razón de tasas, respectivamente ^22^.

Estas medidas de heterogeneidad se interpretan como el cambio relativo mediano en la medida de ocurrencia del evento, cuando se compara el resultado de un individuo de un grupo seleccionado al azar, con el de otro individuo con valores covariados idénticos, pero seleccionado al azar de un grupo diferente, de un conjunto de grupos ordenados por dicha medida ^22^. Indican el cambio en la probabilidad individual respecto a un resultado al cambiar de un grupo de menor riesgo a otro de mayor riesgo. Por ejemplo, una mediana de la *Odds Ratio* igual a 1 indica que no habría diferencias entre grupos en la probabilidad de presentar el resultado. Si hubiese fuertes diferencias entre grupos, dicha mediana sería grande y la variable de agrupación sería relevante para comprender las variaciones en la probabilidad individual de determinado resultado ^23^.

Ballesteros y Moreno-Montoya ^24^ utilizaron un modelo *logit* de dos niveles para identificar los principales factores regionales asociados con variaciones en la prevalencia de limitación funcional en el adulto mayor en Colombia. Tras ajustar por variables, se calculó la mediana de la *Odds Ratio* para evaluar en qué medida la probabilidad individual de limitación funcional estaba determinada por las variables a nivel de estado. El análisis mostró diferencias estadísticamente significativas en la prevalencia de limitación funcional entre los estados, particularmente, en cuanto al nivel socioeconómico medido según el Índice de Desarrollo Humano (MOR=1,22; IC_95%_: 1,13-1,30).

## Pasos generales para la construcción de un modelo de regresión multinivel

Para plantear un análisis de regresión multinivel, primero se requiere revisar la teoría existente, las investigaciones previas y el análisis exploratorio de los datos, con el fin de comprobar la existencia de una estructura jerárquica ^25^. Al igual que en las regresiones convencionales, es conveniente postular con anterioridad los predictores por considerar en las diferentes fases del análisis, lo mismo que sus niveles ^26^ y el número de sujetos por incluir en cada nivel, ya que el poder depende tanto del número de grupos como del número de unidades en cada grupo ^2^. También, es necesario asegurar la medición de las características a nivel grupal, si se sospecha que los individuos pertenecen a grupos en los cuales comparten dichas características ^27^.

Estadísticamente, primero es necesario validar la idoneidad de un análisis multinivel. Esto es posible mediante el cálculo de la proporción de la varianza total, explicada por características de niveles superiores por medio del coeficiente de correlación intraclase (CCI) que en un modelo multinivel de efectos fijos, corresponde al mismo coeficiente de partición de la varianza (CPV) ^28^:




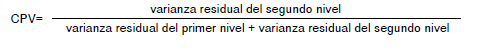




Para el análisis del coeficiente, se debe considerar que este toma valores entre 0 y 1. Un valor de cero (0) indica la inexistencia de diferencia entre las observaciones debida a la agrupación (toda la variabilidad de la variable dependiente está explicada por características del primer nivel). Un valor de uno ^1^ indica que no hay diferencias dentro del grupo (toda la variabilidad de la variable dependiente está explicada por características del segundo nivel) ^28^. Cuanto más grande el valor del CCI, más conveniente resulta proceder con el análisis multinivel ^29^, pues habrá un mejor indicador de la existencia y la necesidad de considerar la estructura anidada en los modelos analíticos ^27^^,^^30^.

El CCI también es importante en relación con otros aspectos del análisis multinivel, como el cálculo del tamaño de muestra efectivo. Las muestras agrupadas no son tan eficientes estadísticamente como las muestras aleatorias simples, debido a que las similitudes entre los sujetos de los conglomerados pueden reducir la variabilidad de las respuestas de un conglomerado en comparación con las esperadas de una muestra aleatoria simple, lo que puede agrandar las diferencias entre los grupos. Esta condición implica que, al ajustar el tipo de muestreo, se reduzca el tamaño efectivo de la muestra y, por tanto, la precisión en las estimaciones:




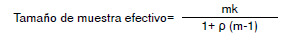




En esta fórmula, m es el número de grupos, k es número de unidades por grupo y p es el CCI. El aumento del CCI o el número de unidades por grupo, implican un menor tamaño de muestra efectiva y menor potencia, mientras que una mayor cantidad de grupos puede implicar un mayor tamaño de muestra efectiva y mayor potencia ^31^.

Respecto a los resultados de un modelo multinivel, como se mencionó previamente, el CCI o el CPV también pueden ser utilizados para reportar la medida del efecto contextual general, es decir, la cantidad de variabilidad que aporta el efecto de agrupación con respecto a la cantidad de variabilidad del fenómeno *per se* ^27^^,^^30^.

A continuación, se proponen algunos pasos generales y usualmente utilizados para guiar el planteamiento de un modelo de regresión multinivel, una vez se ha confirmado teórica y estadísticamente que se puede utilizar, y se ha definido el modelo estadístico, según la pregunta de investigación y la estructura de los datos.

## Generar un modelo nulo

Este tiene una finalidad descriptiva, pretende servir como punto de referencia para los modelos que siguen ^32^. Se utiliza para analizar la variabilidad de la variable dependiente dentro y entre los niveles superiores ^33^. Además, sirve para valorar la magnitud de la variación que depende de los promedios formados por los grupos sin considerar los efectos de las variables explicativas.

La ecuación del modelo nulo sin variables explicativas se expresa como




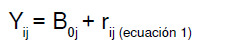




Y_ij_: valor para Y del individuo i en el grupo j 

B_0j_: promedio de Y en el grupo j

r_ij_: efecto aleatorio de los individuos i en el grupo j. Diferencia entre el valor de Y para cada i en el grupo j y el promedio de Y en el grupo j (B_oj_).

El promedio de Y en el grupo j, a su vez, se calcula como




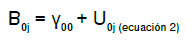




Y_00_: promedio general de todos los grupos en los niveles superiores

U_0j_: efecto aleatorio de los grupos. Diferencia entre el promedio de cada grupo y el promedio general de todos los grupos en los niveles superiores (y_00_)

El modelo nulo de los dos niveles es




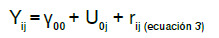




Hasta este momento, no se tienen variables predictoras y es posible identificar un componente fijo (y_00_)y un componente aleatorio (U_0j_ + e_ij_). El componente fijo corresponde a la media poblacional y no cambia entre grupos, mientras que el componente aleatorio corresponde a los residuales de los dos niveles y puede variar de un grupo a otro ^32^.

**Figura 2:**
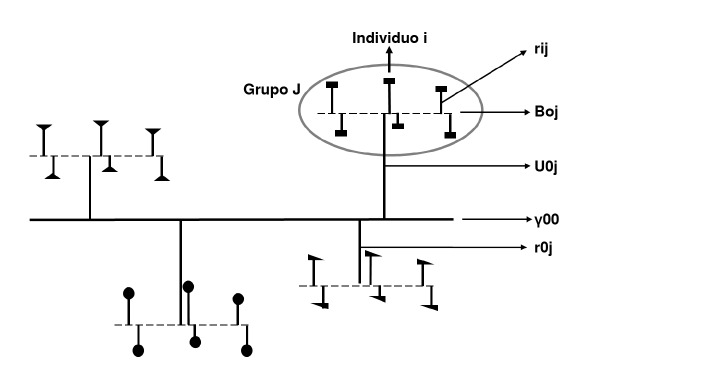
Elementos incluidos en el modelo nulo

Cada uno de los elementos de la ecuación del modelo nulo se puede identificar gráficamente en la figura 2.

## Modelo multinivel general de dos niveles

Para la estimación de un modelo de dos niveles, se deben considerar dos ecuaciones: la primera corresponde al nivel individual, para determinar la variación individual dentro de cada grupo (2).

Ecuación del modelo a nivel individual




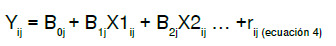




Y_ij_: valor para Y del individuo i en el grupo j

B_0j_: promedio de Y en el grupo j

B_1j_: coeficiente del nivel 1

X_ij_: predictor del nivel 1 para el individuo i en el grupo j

r_ij_: efecto aleatorio del nivel 1

La segunda ecuación considera la variación de los coeficientes entre grupos ^2^. Los coeficientes B_0j_ y B_1j_ se convierten en una variable dependiente y pueden ser aleatorios, dependiendo del objetivo del análisis ^32^.

Ecuación del modelo a nivel de grupo para B_0j_




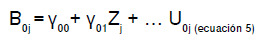




B_0j_: intersección del primer nivel

γ_00_: promedio general de todos los grupos en los niveles superiores

γ_01_: pendiente del segundo nivel

Z_j_: predictor del segundo nivel

U_0j_: efecto aleatorio del nivel dos. Mide la desviación de la intersección de cada grupo respecto a la intersección general, después de ajustar por Z_j_^2^.

Ecuación del modelo a nivel de grupo para B_1j_




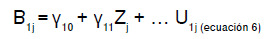




B_1j_: pendiente del primer nivel

γ_10_: pendiente general

γ_11_: pendiente del segundo nivel

Z_j_: predictor del segundo nivel

U_1j_: Efecto aleatorio del nivel dos. Mide la desviación de la pendiente de cada grupo respecto a la pendiente general, después de ajustar por Z_j_^2^.

Con base en las especificaciones anteriores, se presentan las ecuaciones de los modelos: a) solo con variable independiente individual (ecuación 7), b) solo con variable independiente grupal (ecuación 8), y c) con variables independientes individual y grupal (ecuación 9).




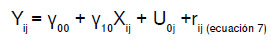







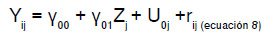







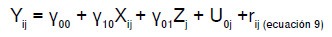




Finalmente, se presenta la ecuación para especificar un modelo de efectos aleatorios (intercepto y pendiente aleatoria). El modelo incluye los efectos fijos de las variables del segundo nivel (Y_01_), las variables del primer nivel (y_10_) y su interacción (Y_11_) sobre el resultado a nivel individual Y También, incluye los componentes aleatorios de la intersección (U_0j_), la pendiente U_1j_ y el primer nivel (r_ij_) 2.

Ecuación del modelo de efectos aleatorios




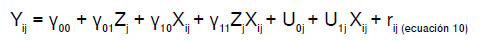




**Figura 3:**
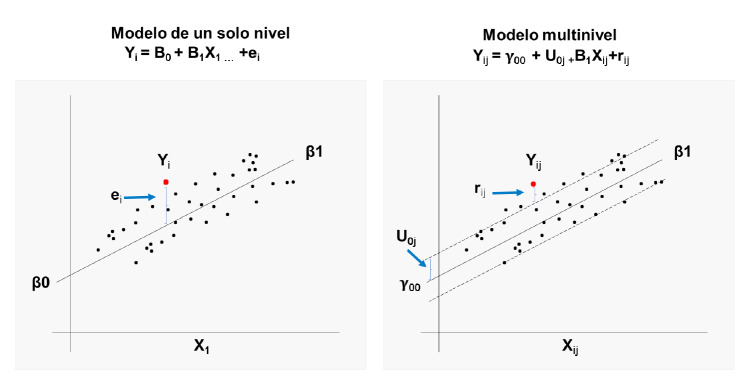
Componentes fijos y aleatorios del modelo multinivel comparado con un modelo de un solo nivel

La figura 3 presenta los componentes fijos y aleatorios del modelo multinivel comparado con un modelo de un solo nivel.

Tanto en el modelo con un nivel como en el de dos niveles, se debe especificar y reportar si los predictores han sido centrados en algún nivel específico porque esto es clave en el momento de interpretar los resultados ^25^. Según Peugh, el centrado implica "volver a escalar una variable predictora para que un valor de cero pueda interpretarse de manera significativa", y esto es particularmente útil cuando se utilizan variables predictoras de tipo ordinal en las que el cero no tiene un valor ^27^.

## Evaluación del ajuste del modelo final

Para evaluar y seleccionar el modelo con mejor ajuste, se han propuesto dos técnicas principales: el análisis de la razón de verosimilitud y la evaluación mediante índices de criterio de información, como el criterio de información de Akaike *(Akaike Information Criterion,* AIC) y el criterio de información bayesiano *(Bayesian Information Criterion,* BIC) ^25^. Estos dos últimos procuran evitar el ajuste excesivo que puede generar la agregación de parámetros al modelo e incluyen un término de penalización para el número de parámetros ^34^.

La razón de verosimilitud *(likelihood ratio)* o *deviance,* tiene una distribución x^2^ con grados de libertad iguales a la diferencia de parámetros de los modelos anidados ^33^, y se calcula como


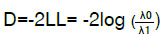
 , donde λ_0_ corresponde al valor de verosimilitud *(likelihoods)* del modelo sin predictores y λ _1_ del modelo final ^35^. La prueba sugiere que a menor valor, mejor ajuste ^33^.

Según el AIC, el mejor modelo es aquel que explica la mayor cantidad de variación, utilizando la menor cantidad posible de variables independientes, y se calcula como AIC =-2LL+2q, siendo *q* el número de parámetros estimados ^36^. El mejor modelo será aquel con los valores de AIC más bajos, pero los valores numéricos que arroja su cálculo no tienen un significado ^37^.

Por su parte, el BIC se calcula como BIC=-2LL+ln(N)q, siendo *N* el número de muestras del nivel 1 ^36^. Este criterio aumenta la penalización cuando aumenta el tamaño de muestra y, por tanto, comparado con el AIC, es más difícil lograr la significancia ^34^. También, se considera que el modelo con mejor ajuste es aquel con BIC más bajo (Alvarado WR. Aplicación de la teoría de modelos multinivel lineal y no-lineal utilizando el *software* especializado HLM7. En: X Congreso Internacional sobre la enseñanza de la matemática asistida por computadora. Tecnológico de Costa Rica. 2017. Disponible en: https://www.tec.ac.cr/sites/default/files/media/doc/10_memoria_1.pdf).

En cuanto a los *softwares* para la implementación de análisis multinivel, existen programas estadísticos como MLwiN ^38^, diseñados específicamente para estimar estos modelos, y otros como R que son programas de análisis estadístico general que han incluido paquetes y procesos para el análisis de datos anidados. Albright y Marinova ^39^ presentan una guía sobre cómo estimar modelos multinivel utilizando SPSS, Stata, SAS y R.

## Experiencias en investigación en salud con análisis multinivel

El análisis multinivel se ha utilizado para dar respuesta a preguntas de investigación relacionadas con resultados que dependen de factores individuales, ambientales y sociales, es decir, que no dependen del sujeto en sí mismo, sino también de su interacción con el entorno y con los demás ^5^. A continuación, se describen algunos estudios que, a juicio de los autores, pueden ilustrar la aplicación de estos modelos en la investigación sanitaria (cuadro 1).

**Cuadro 1:** Ejemplos de aplicación del análisis multinivel en investigación en salud

Estudio	Población y estructura	Modelos	Resultados	Análisis complementario
Asociaciones entre los factores a nivel de vecindario y la mortalidad relacionada con los opioides: un análisis multinivel utilizando datos de certificados de defunción. (Flores *et al*., 2020) ^40^	Estructura multinivel clásica **Nivel 1:** individuos; 3.809 muertes prematuras relacionadas con opioides y 8.729 no relacionadas con opioides **Nivel 2:** 2.517 grupos de bloques censales **Nivel 3:** 14 condados	**Modelo 1:** de intersecciones aleatorias sin covariables **Modelo 2:** con datos a nivel individual y de área	Algunos resultados: Hay mayor mortalidad a mayor porcentaje de personas en la pobreza (OR=1,01, IC_95%_: 1,00- 1,01) y a mayor tasa de inseguridad alimentaria (OR=1,21, IC_95%_: 1,07- 1,37). Hay menor mortalidad a mayor número de camas de hospital por 10.000 personas (OR=0,78, _95%_: 0,68-0,88).	Se realizaron tres análisis de sensibilidad: en los dos primeros, se identificaron hallazgos similares a los del modelo principal; en el tercero, se agregaron secuencialmente categorías de factores de riesgo a nivel de área y como resultado se observaron varias asociaciones que no eran frecuentes en el modelo principal.
La satisfacción con la relación predice un menor estrés e inflamación en sobrevivientes de cáncer de mama: un estudio longitudinal de los efectos dentro de la persona y entre personas. (Shrout *et al.*, 2020) ^41^.	Medidas repetidas **Nivel 1:** tres mediciones, antes, durante y después del tratamiento **Nivel 2:** 139 participantes recibieron tres visitas.	Modelos multinivel para proteína C reactiva (PCR) sérica y compuesto de citosinas 1. Intrapersonal 2. Interpersonal	Respecto a los resultados relacionados con la PCR a nivel intrapersonal: a mayor satisfacción en la relación, menor estrés percibido (β = 0,19*). Mayor estrés se relacionó con niveles más altos de PCR (β = 0,55**). La satisfacción con la relación no se asoció directamente con la PCR, pero sí indirectamente por medio estrés percibido (β = -0,10*).	Modelos hipotéticos para explorar si el estrés estaba indirectamente relacionado con la inflamación por medio de la satisfacción con la relación tuvieron peor ajuste que los modelos planteados inicialmente: AIC = 1.825,72 versus 1.954,36 para PCR. AIC = 2.064,40 versus 2.192,36 del modelo compuesto por citocinas.
Modelos multinivel de clasificación cruzada para la gravedad de los accidentes automovilísticos comerciales teniendo en cuenta la heterogeneidad entre empresas y regiones. (Park *et al*., 2017) ^42^.	Clasificación cruzada **Nivel 1:** 86.622 choques de vehículos **Nivel 2:** dos grupos no anidados; 1.875 empresas y 230 municipios	**Modelo 1:** un solo nivel **Modelo 2:** dos niveles, uno para empresa y otro para región **Modelo 3:** combinación de las variables empresa y región Cada modelo se planteó para cuatro tipos de transporte. En total, se generaron 12 modelos, la mayoría con CCI >12,1 %.	Algunos hallazgos incluyen: Consecuencias menos graves cuando el conductor era el dueño del taxi (β = 0,490*). En municipios con presupuestos de infraestructura de transporte relativamente grandes, hubo menos accidentes graves para camiones grandes (-0,0139**), autobuses (-0,008**), y taxis (-0,005**).	No se reporta.

*p<0,05, **p<0,01; AIC: criterio de información de Akaike; CCI: coeficiente de correlación intraclase

Flores *et al.* ^40^ plantearon un análisis multinivel exploratorio para identificar asociaciones entre la mortalidad relacionada con los opioides y los factores de riesgo a nivel de vecindario en Massachusetts (Estados Unidos), en busca de información que aportara en la planeación de intervenciones poblacionales y partiendo del hecho de que, según la evidencia disponible hasta el momento, las intervenciones individuales y del lado de la oferta eran insuficientes. Plantearon una estructura multinivel clásica de individuos anidados en bloques censales y estos, a su vez, en condados (equivalente a municipios para Colombia). Para el análisis estadístico, se propusieron modelos de regresión logística multinivel de efectos fijos (interceptos aleatorios), debido a que solo estaban interesados en conocer el efecto de las variables contextuales. Encontraron que, en la población de estudio, las muertes relacionadas con opioides podrían estar asociadas positivamente con el porcentaje de personas que vivían en la pobreza, la tasa de inseguridad alimentaria, el número de centros de salud calificados a nivel federal y los miligramos equivalentes de hidromorfona per cápita, pero inversamente relacionados con el número de asociaciones sociales por 10.000 habitantes y el número de camas de hospital por 10.000 habitantes.

Por su parte, Shrout *et al.* ^41^. evaluaron los efectos dentro y entre personas de la satisfacción con las relaciones románticas sobre la inflamación (marcadores séricos) por medio del estrés psicológico percibido en sobrevivientes de cáncer de mama. Midieron las variables de interés antes del tratamiento, y a los 6 y 18 meses después de finalizar el tratamiento. Plantearon un análisis de mediación que pretende comprender cómo una variable independiente produce un efecto indirecto sobre un resultado por medio de una variable interviniente o mediadora. Dado que los datos tenían una estructura jerárquica con individuos en el nivel 2 (individuos) y medidas repetidas (no independientes) de cada individuo en el nivel 1, realizaron un análisis de mediación multinivel. En el estudio, se concluyó que, tanto en el análisis intrapersonal como en el interpersonal, una mejor satisfacción con la relación está asociada con una menor percepción del estrés, lo que, a su vez, se relaciona con menores niveles de inflamación.

Park *et al.* ^42^. evaluaron los factores que afectan la gravedad de los accidentes de vehículos comerciales de motor. Los accidentes fueron clasificados en cuatro grupos: mortal, mayor, menor y sin lesiones. Se incluyeron 865.622 accidentes. Consideraron variables a nivel individual y variables a nivel de grupos no anidados (empresas y municipios). Dada la estructura de los datos, para el análisis de la información plantearon cuatro modelos: un modelo *logit* de un solo nivel, dos modelos *logit* multinivel convencionales y un modelo *logit* multinivel con clasificación cruzada. Identificaron una relación inversa y estadísticamente significativa entre la proporción del presupuesto de infraestructura de transporte en el municipio, y la gravedad de las muertes y las lesiones de todos los tipos de accidentes.

Finalmente, si bien la presente revisión pretende abordar los elementos fundamentales del uso clásico del análisis multinivel, es importante señalar que hay evidencia emergente sobre usos específicos como su aplicación en salud pública. Evans *et al.* ^42^^) (^^43^ plantearon un análisis multinivel de heterogeneidad individual y precisión discriminatoria que consiste en utilizar modelos jerárquicos multinivel en el marco de la interseccionalidad. Esta percepción crítica sugiere que múltiples categorías sociales no son atributos individuales, sino sistemas interrelacionados de opresión que interactúan desde lo individual hasta lo estructural. En este ámbito, el análisis multinivel permite capturar las desigualdades en salud en diferentes estratos interseccionales de la población ^44^.

En este trabajo, se resumen de forma preliminar el alcance y la utilidad de los modelos estadísticos multinivel en el ámbito de la investigación en salud.

Si bien esta metodología cuenta con varias décadas desde su aparición en publicaciones científicas masivas, prevalece todavía algún rezago para su aplicación debido a las consideraciones estadísticas y técnicas necesarias. Por esta razón, este documento presenta una breve aproximación a las consideraciones que deben tomarse en cuenta para su planteamiento y su construcción. Así mismo, se señalan algunos ejemplos que pueden ilustrar su aplicación.

El alcance de esta metodología, más allá de su capacidad para ponderar el efecto de una serie de variables sobre un resultado particular, radica en la posibilidad de integrar información con estructuras complejas, para ahondar en la comprensión de la trama de interacciones y relaciones que a nivel social, clínico y biológico confluyen en la aparición de condiciones de salud; y para plantear estrategias e iniciativas que, desde una mirada más amplia, incrementen los aspectos por considerar en el momento de promover acciones sanitarias benéficas.

Si bien los modelos siguen requiriendo sendos conocimientos estadísticos, la existencia de *softwares* como MLwiN facilitan la tarea de su construcción, a la vez que llaman la atención sobre su uso riguroso. Bancos de información con estructuras más complejas de anidamiento, frecuencias bajas de ocurrencia en el resultado de interés, desequilibrio en la cantidad de información disponible, entre otras situaciones, constituyen múltiples desafíos para el uso de esta metodología. Sin embargo, dado su aporte en la extensión de los modelos de regresión convencionales, el modelamiento multinivel se convierte en uno de los pilares fundamentales por fortalecer en lo concerniente a la indagación cuantitativa en salud. Su uso no debería depender de los gustos particulares del investigador, sino de la necesidad de explicar efectos de manera independiente en grupos que naturalmente se aglomeran, lo cual resalta su importancia en el estudio de problemas de salud pública.

## References

[1] De la Cruz F (2008). Modelos multinivel. Rev Per Epidemiol.

[2] Diez-Roux AV (2000). Multilevel analysis in public health research. Annu Rev Public Health.

[3] Usami S (2014). Generalized sample size determination formulas for experimental research with hierarchical data. Behav Res Methods.

[4] Rasbash J, Steele F, Browne WJ, Goldstein H University of Bristol, Centre for Multilevel Modelling MLwiN, version 3.0.

[5] Diez-Roux AV (2008). La necesidad de un enfoque multinivel en epidemiología. Region Soc.

[6] Fisher AJ, Medaglia JD, Jeronimus BF (2018). Lack of group-to-individual generalizability is a threat to human subjects research. Proc Natl Acad Sci USA.

[7] University of Bristol Centre for Multilevel Modelling. What are multilevel models and why should I use them?.

[8] Damtie Y, Kefale B, Yalew M, Arefaynie M, Adane B (2021). Multilevel analysis of determinants of polygyny among married men in Ethiopia. BMC Glob Public Health.

[9] Dessie ZG, Zewotir T, Mwambi H, North D (2020). Multivariate multilevel modeling of quality of life dynamics of HIV infected patients. Health Qual Life Outcomes.

[10] Hagadorn JI, Shaffer ML (2019). Hierarchical data structures and multilevel modeling. J Pediatr.

[11] Huang F (2018). Multilevel modeling myths. Sch Psychol Q.

[12] Ntani G, Inskip H, Osmond C, Coggon D (2021). Consequences of ignoring clustering in linear regression. BMC Med Res Methodol.

[13] Mumper M. (2017). American Psycological Association.

[14] University of Bristol Centre for Multilevel Modelling. Multilevel models: An introduction and FAQs.

[15] Barker KM, Dunn EC, Richmond TK, Ahmed S, Hawrilenko M, Evans CR (2020). Cross-classified multilevel models (CCMM) in health research: A systematic review of published empirical studies and recommendations for best practices. SSM Popul Health.

[16] Finch H, Bolin JE, Kelley K (2019). Multilevel modeling using R.

[17] University of Bristol Centre for Multilevel Modelling. Random intercept models.

[18] Speed TP, Balakrishnan N, Colton T, Everitt B, Piegorsch W, Ruggeri F, Teugels JL (2014). Wiley StatsRef: Statistics Reference Online.

[19] Bolin JH, Finch WH, Stenger R (2019). Estimation of random coefficient multilevel models in the context of small numbers of level 2 clusters. Educ Psychol Meas.

[20] Webster TF, Hoffman K, Weinberg J, Vieira V, Aschengrau A (2008). Community- and individuallevel socioeconomic status and breast cancer risk: Multilevel modeling on Cape Cod, Massachusetts. Environ Health Perspect.

[21] Austin PC, Wagner P, Merlo J (2017). The median hazard ratio: A useful measure of variance and general contextual effects in multilevel survival analysis. Stat Med.

[22] Austin PC, Stryhn H, Leckie G, Merlo J (2018). Measures of clustering and heterogeneity in multilevel Poisson regression analyses of rates/count data. Stat Med.

[23] Merlo J, Chaix B, Ohlsson H, Beckman A, Johnell K, Hjerpe P (2006). A brief conceptual tutorial of multilevel analysis in social epidemiology: Using measures of clustering in multilevel logistic regression to investigate contextual phenomena. J Epidemiol Community Health.

[24] Ballesteros SM, Moreno-Montoya J (2018). Factores individuales y departamentales asociados con la prevalencia de limitación funcional entre ancianos colombianos: un análisis multinivel. Cad Saúde Pública.

[25] Dedrick RF, Ferron JM, Hess MR, Hogarty KY, Kromrey JD, Lang TR (2009). Multilevel modeling: A review of methodological issues and applications. Rev Educ Res.

[26] Catalán-Reyes MJ, Galindo-Villardón MP (2003). Utilización de los modelos multinivel en investigación sanitaria. Gac Sanit.

[27] Peugh JL (2010). A practical guide to multilevel modeling. J Sch Psychol.

[28] Osorio AM, Romero GA, Bonilla H, Aguado LF (2018). Socioeconomic context of the community and chronic child malnutrition in Colombia. Rev Saúde Pública.

[29] Gabriëlle I, Jongmans M (2021). Intra-class correlation testing to examine Intra-group differences.

[30] Yamana H (2021). Introduction to multilevel analysis. Ann Clin Epidemiol.

[31] Killip S, Mahfoud Z, Pearce K (2004). What is an intracluster correlation coefficient? Crucial concepts for primary care researchers. Ann Fam Med.

[32] Ramos-Rodríguez FJ, Lara Porras AM, Molina-Muñoz D. (2019). Competencia matemática de los estudiantes andaluces: un análisis multinivel de la encuesta PISA 2015. Pi-InnovaMath.

[33] Alarcón R, Blanca MJ, Arnau J, Bono R (2015). Modelado jerárquico por pasos: análisis multinivel del estrés cotidiano en adolescentes. Rev Mex Psicol.

[34] Vrieze SI (2012). Model selection and psychological theory: A discussion of the differences between the Akaike Information Criterion (AIC) and the Bayesian Information Criterion (BIC). Psychol Methods.

[35] Oliver JC, Rosel J, Jara P (2000). Modelos de regresión multinivel: aplicación en psicología escolar. Psicothema.

[36] Kim S, Jeong Y, Hong S (2021). The impact of ignoring a crossed factor in cross-classified multilevel modeling. Front Psychol.

[37] Portet S (2020). A primer on model selection using the Akaike Information Criterion. Infect Dis Model.

[38] University of Bristol (2022). Centre for Multilevel Modelling.

[39] Albright JJ, Marinova DM (2010). Estimating multilevel models using SPSS. Stata.

[40] Flores MW, Cook BL, Mullin B, Halperin-Goldstein G, Nathan A, Tenso K (2020). Associations between neighborhood-level factors and opioid-related mortality: A multilevel analysis using death certificate data. Addict Abingdon Engl.

[41] Shrout MR, Renna ME, Madison AA, Alfano CM, Povoski SP, Lipari AM (2020). Relationship satisfaction predicts lower stress and inflammation in breast cancer survivors: A longitudinal study of within-person and between-person effects. Psychoneuroendocrinology.

[42] Park HC, Kim DK, Kho SY, Park PY (2017). Cross-classified multilevel models for severity of commercial motor vehicle crashes considering heterogeneity among companies and regions. Accid Anal Prev.

[43] Evans CR, Williams DR, Onnela JP, Subramanian SV (2018). A multilevel approach to modeling health inequalities at the intersection of multiple social identities. Soc Sci Med.

[44] Arias-Uriona AM, Losantos M, Bedoya P (2023). La interseccionalidad como herramienta teóricoanalítica para estudiar las desigualdades en salud en las Américas. Rev Panam Salud Pública.

